# Supplementary material for: The efficacy and safety of sodium tanshinone ⅡA sulfonate injection in the treatment of unstable angina pectoris: A systematic review and meta-analysis
Source: PLoS One. 2023 Aug 31;18(8):e0290841. doi: 10.1371/journal.pone.0290841 (PMC10470971; doi:10.1371/journal.pone.0290841)
Supplement: Attachment — Submitted filename: PONE-D-23-09981_reviewer.pdf [file pone.0290841.s002.pdf]

PLOS ONE

The efficacy and safety of Sodium tanshinone IIA sulfonate injection in the treatment of unstable angina pectoris: a systematic review and meta-analysis

--Manuscript Draft--

**Manuscript Number:**	PONE-D-23-09981
**Article Type:**	Research Article
**Full Title:**	The efficacy and safety of Sodium tanshinone IIA sulfonate injection in the treatment of unstable angina pectoris: a systematic review and meta-analysis
**Short Title:**	Sodium tanshinone IIA sulfonate injection in the treatment of unstable angina pectoris
**Corresponding Author:**	Dong Guo<br>Shandong University of Traditional Chinese Medicine<br>CHINA
**Keywords:**	Keywords: Sodium tanshinone IIA sulfonate injection; Unstable angina pectoris; System review; meta-analysis.
**Abstract:**	Abstract<br>Objective To systematically evaluate the efficacy and safety of Sodium tanshinone IIA sulfonate injection (STS) in the treatment of unstable angina pectoris (UAP). Methods CNKI, Wanfang, VIP, CBM, PubMed, Cochrane Library, Web of Science, Embase were searched by computer. The time was the clinical randomized controlled trials of STS in the treatment of unstable angina pectoris published from the establishment of the library to January 31, 2023. Two researchers independently screened the literature, extracted data and evaluated the risk of research bias, and then conducted meta-analysis with RevMan5.3 software.<br>Results A total of 37 randomized controlled trials were included, with 3926 patients in total. Meta analysis results showed that STS combined with conventional western medicine could reduce the frequency of angina pectoris(SMD=-2.61, 95%CI[-4.27, -0.96], P=0.002) and the duration of angina pectoris(SMD=-4.01, 95%CI[-6.18, -1.84], P=0.0003), improve ECG efficacy(OR=3.61, 95%CI[2.79, 4.68], P < 0.00001) and clinical symptom efficacy(OR=4.02, 95%CI[3.32, 4.87], P < 0.00001), reduce TG(SMD=-0.60, 95%CI[-1.04, -0.16], P=0.008), TC(SMD=-3.86, 95%CI[-6.37, -1.34], P=0.003), LDL-C(SMD=-1.54, 95%CI[-2.67, -0.42], P=0.007), reduce plasma viscosity(SMD=-1.02, 95%CI[-1.58, -0.47], P < 0.0003), whole blood low shear viscosity(SMD=-0.85, 95%CI[-1.21, -0.49], P < 0.00001), whole blood high shear viscosity(SMD=-0.82, 95%CI[-1.44, -0.20], P=0.009), erythrocyte aggregation index(SMD=-1.00, 95%CI[-1.75, -0.25], P=0.009), reduce CRP(SMD=-1.39, 95%CI[-1.91, -0.86], P<0.00001) was better than that of conventional western medicine alone. The incidence of adverse reactions in the treatment group was higher than that in the control group (OR=2.26, 95%CI[1.06, 4.85], P=0.04). Neither of the two groups affected the liver and kidney function, nor the study process.<br>Conclusion STS combined with conventional western medicine therapy has a definite clinical efficacy and certain safety in the treatment of UAP, but it needs to be further confirmed by high-quality and low bias risk randomized controlled trials in the future.
**Order of Authors:**	Xiaoqi Wu
	Maoxia Fan
	Sining Wei
	Dong Guo
**Additional Information:**	
**Question**	**Response**
**Financial Disclosure**<br><br>Enter a financial disclosure statement that describes the sources of funding for the	The funders had no role in study design, data collection and analysis, decision to publish, or preparation of the manuscript.

Powered by Editorial Manager® and ProduXion Manager® from Aries Systems Corporation

work included in this submission. Review the submission guidelines for detailed requirements. View published research articles from *PLOS ONE* for specific examples.<br><br>This statement is required for submission and **will appear in the published article** if the submission is accepted. Please make sure it is accurate.<br><br>**Unfunded studies**<br>Enter: *The author(s) received no specific funding for this work.*<br><br>**Funded studies**<br>Enter a statement with the following details:<br>• Initials of the authors who received each award<br>• Grant numbers awarded to each author<br>• The full name of each funder<br>• URL of each funder website<br>• Did the sponsors or funders play any role in the study design, data collection and analysis, decision to publish, or preparation of the manuscript?<br>• **NO** - Include this sentence at the end of your statement: *The funders had no role in study design, data collection and analysis, decision to publish, or preparation of the manuscript.*<br>• **YES** - Specify the role(s) played.<br><br>* typeset	
**Competing Interests**<br><br>Use the instructions below to enter a competing interest statement for this submission. On behalf of all authors, disclose any competing interests that could be perceived to bias this work—acknowledging all financial support and any other relevant financial or non-financial competing interests.<br><br>This statement is required for submission and **will appear in the published article** if the submission is accepted. Please make sure it is accurate and that any funding sources listed in your Funding Information	The authors have declared that no competing interests exist.

later in the submission form are also declared in your Financial Disclosure statement.<br><br>View published research articles from *PLOS ONE* for specific examples.<br><br>**NO authors have competing interests**<br><br>Enter: *The authors have declared that no competing interests exist.*<br><br>**Authors with competing interests**<br><br>Enter competing interest details beginning with this statement:<br><br>*I have read the journal's policy and the authors of this manuscript have the following competing interests: [insert competing interests here]*<br><br>* typeset	
**Ethics Statement**<br><br>Enter an ethics statement for this submission. This statement is required if the study involved:<br><br>• Human participants<br>• Human specimens or tissue<br>• Vertebrate animals or cephalopods<br>• Vertebrate embryos or tissues<br>• Field research<br><br>Write "N/A" if the submission does not require an ethics statement.<br><br>General guidance is provided below. Consult the submission guidelines for detailed instructions. **Make sure that all information entered here is included in the Methods section of the manuscript.**	N/A

**Format for specific study types**<br><br>**Human Subject Research (involving human participants and/or tissue)**<br>• Give the name of the institutional review board or ethics committee that approved the study<br>• Include the approval number and/or a statement indicating approval of this research<br>• Indicate the form of consent obtained (written/oral) or the reason that consent was not obtained (e.g. the data were analyzed anonymously)<br><br>**Animal Research (involving vertebrate animals, embryos or tissues)**<br>• Provide the name of the Institutional Animal Care and Use Committee (IACUC) or other relevant ethics board that reviewed the study protocol, and indicate whether they approved this research or granted a formal waiver of ethical approval<br>• Include an approval number if one was obtained<br>• If the study involved *non-human primates*, add *additional details* about animal welfare and steps taken to ameliorate suffering<br>• If anesthesia, euthanasia, or any kind of animal sacrifice is part of the study, include briefly which substances and/or methods were applied<br><br>**Field Research**<br><br>Include the following details if this study involves the collection of plant, animal, or other materials from a natural setting:<br>• Field permit number<br>• Name of the institution or relevant body that granted permission	
**Data Availability**<br><br>Authors are required to make all data underlying the findings described fully available, without restriction, and from the time of publication. PLOS allows rare exceptions to address legal and ethical concerns. See the PLOS Data Policy and FAQ for detailed information.	Yes - all data are fully available without restriction

A Data Availability Statement describing where the data can be found is required at submission. Your answers to this question constitute the Data Availability Statement and **will be published in the article**, if accepted.<br><br>**Important:** Stating 'data available on request from the author' is not sufficient. If your data are only available upon request, select 'No' for the first question and explain your exceptional situation in the text box.<br><br>Do the authors confirm that all data underlying the findings described in their manuscript are fully available without restriction?	
**Describe where the data may be found in full sentences. If you are copying our sample text, replace any instances of XXX with the appropriate details.**<br><br>• If the data are **held or will be held in a public repository**, include URLs, accession numbers or DOIs. If this information will only be available after acceptance, indicate this by ticking the box below. For example: *All XXX files are available from the XXX database (accession number(s) XXX, XXX.*).<br>• If the data are all contained **within the manuscript and/or Supporting Information files**, enter the following: *All relevant data are within the manuscript and its Supporting Information files.*<br>• If neither of these applies but you are able to provide **details of access elsewhere**, with or without limitations, please do so. For example:<br><br>*Data cannot be shared publicly because of [XXX]. Data are available from the XXX Institutional Data Access / Ethics Committee (contact via XXX) for researchers who meet the criteria for access to confidential data.*<br><br>*The data underlying the results presented in the study are available from (include the name of the third party*	All relevant data are within the manuscript and its Supporting Information files.

and contact information or URL).<br>• This text is appropriate if the data are owned by a third party and authors do not have permission to share the data.<br><br>* typeset	
Additional data availability information:	Tick here if your circumstances are not covered by the questions above and you need the journal’s help to make your data available.

Cover Letter

Click here to access/download;Manuscript;4.3 TST for UAP.docx

The efficacy and safety of Sodium tanshinone ⅡA sulfonate injection in the treatment of unstable angina pectoris: a systematic review and meta-analysis

Xiaoqi Wu[1] , Maoxia Fan[1],Sining Wei[2],Dong Guo[*]

[1]Xiaoqi Wu Shandong University of Traditional Chinese Medicine, Jinan, People's Republic of China Email 2020111279@sdutcm.edu.cn

[1]Maoxia Fan Shandong University of Traditional Chinese Medicine, Jinan, People's Republic of China

[2]Sining Wei Affiliated Hospital of Shandong University of Traditional Chinese Medicine, Jinan, People's Republic of China

[*]Correspondence:

Dong Guo, Email qlyp@sdutcm.edu.cn.

Shandong University of Traditional Chinese Medicine, No.16369, Wendong Street, Lixia District, Jinan City, Shandong Province, China

Abstract

Objective To systematically evaluate the efficacy and safety of Sodium tanshinone ⅡA sulfonate injection (STS) in the treatment of unstable angina pectoris (UAP). **Methods** CNKI, Wanfang, VIP, CBM, PubMed, Cochrane Library, Web of Science, Embase were searched by computer. The time was the clinical randomized controlled trials of STS in the treatment of unstable angina pectoris published from the establishment of the library to January 31, 2023. Two researchers independently screened the literature, extracted data and evaluated the risk of research bias, and then

may mentioned the duration of period

conducted meta-analysis with RevMan5.3 software.

Results A total of 37 randomized controlled trials were included, with 3926 patients in total. Meta analysis results showed that STS combined with conventional western medicine could reduce the frequency of angina pectoris(SMD=-2.61, 95%CI[-4.27, -0.96], *P*=0.002) and the duration of angina pectoris(SMD=-4.01, 95%CI[-6.18, -1.84], *P*=0.0003), improve ECG efficacy(OR=3.61, 95%CI[2.79, 4.68], *P*＜0.00001) and clinical symptom efficacy(OR=4.02, 95%CI[3.32, 4.87], *P*＜0.00001), reduce TG(SMD=-0.60, 95%CI[-1.04, -0.16], *P*=0.008), TC(SMD=-3.86, 95%CI[-6.37, -1.34], *P*=0.003), LDL-C(SMD=-1.54, 95%CI[-2.67, -0.42], *P*=0.007), reduce plasma viscosity(SMD=-1.02, 95%CI[-1.58, -0.47], *P*＜0.0003), whole blood low shear viscosity(SMD=-0.85, 95%CI[-1.21, -0.49], *P*＜0.00001), whole blood high shear viscosity(SMD=-0.82, 95%CI[-1.44, -0.20], *P*=0.009), erythrocyte aggregation index(SMD=-1.00, 95%CI[-1.75, -0.25], *P*=0.009), reduce CRP(SMD=-1.39, 95%CI[-1.91, -0.86], *P*<0.00001) was better than that of conventional western medicine alone. The incidence of adverse reactions in the treatment group was higher than that in the control group (OR=2.26, 95%CI[1.06, 4.85], *P*=0.04). Neither of the two groups affected the liver and kidney function, nor the study process.

Conclusion STS combined with conventional western medicine therapy has a definite clinical efficacy and certain safety in the treatment of UAP, but it needs to be further confirmed by high-quality and low bias risk randomized controlled trials in the future.

Keywords: Sodium tanshinone ⅡA sulfonate injection; Unstable angina pectoris; System review; Meta-analysis.

Introduction

Unstable angina pectoris (UAP) is a common type of angina pectoris, it is caused by coronary artery stenosis, increased blood viscosity, and atheromatous plaque blocking the blood vessels. The main manifestation is that the pain in the anterior chest area is repeated, and the frequency and degree are more severe than before. With the development of the disease, it can cause acute myocardial infarction.[1-2]UAP is currently one of the common chronic diseases that seriously affect the health and quality of life of patients,modern medicine has greatly improved the treatment of this disease,conventional methods include anticoagulation, antiplatelet aggregation drugs β-receptor blockers, nitrates, angiotensin converting enzyme inhibitors, statins and other drugs,[3] but also they face problems such as prominent side effects of drugs and drug dependence.

please standardize the format for Traditional Chinese Medicine

According to its clinical symptoms and signs, Chinese traditional medicine classifies unstable angina pectoris as "chest pain", "heartache", and "Angina

lower case

pectoris".In recent years, with the continuous development of traditional Chinese medicine preparation technology, more and more traditional Chinese medicine preparations have been used to treat unstable angina pectoris, and their efficacy and safety have also received widespread attention.[4]The pharmacological research of

italic

Salvia miltiorrhiza is extensive, and its chemical components are mainly divided into fat-soluble component tanshinone and water-soluble component salvianolic acid. The most widely studied compound in modern times is tanshinone.Sodium tanshinone IIA sulfonate is a substance obtained by chemical modification of the monomer

tanshinone IIA extracted from traditional Chinese medicine Salvia miltiorrhiza,[5]it’s a Chinese herbal extract found in China to treat coronary heart disease.Sodium tanshinone IIA sodium sulfonate has a very good anti-myocardial ischemia and hypoxia effect, can inhibit angiotensin, reduce the occurrence of myocardial remodeling, and improve the clinical symptoms of angina pectoris.[6]Sodium tanshinone ⅡA sulfonate injection (STS) is administered by intravenous drip, which is more conducive to maintaining drug concentration, improving blood circulation, expanding coronary arteries, and reducing heart rate.[7]

There have been frequent reports of randomized controlled clinical trial (RCTs) on the treatment of UAP with STS, but the quality of their studies is low and the conclusions are not completely consistent. Using the method of evidence-based medicine, the effectiveness and safety of STS in the treatment of unstable angina pectoris were systematically evaluated, hoping to provide strong evidence support for clinical research and application.

Material and methods

The methodology of this study followed the Cochrane manual and the report outlined in this study complied with the PRISMA2020 checklist.[8]This system evaluation has been registered on the PROSPERO website with the registration number: PROSPERO CRD42022369967.

2.1 Search strategy

Computer retrieval databases include CNKI, Wanfang, VIP, CBM, PubMed, Cochrane Library, Web of Science, and Embase, included a RCTs of STS in the

treatment of UAP from the establishment of the database to the publication on January 31, 2023.The Chinese search terms included: "coronary heart disease", "angina pectoris", "unstable angina pectoris", "chest pain", "true heartache", "tanshinone IIA sodium sulfonate injection", "tanshinone injection", "randomized", "randomized", "randomized controlled trial"； The English search terms include:"Unstable angina", "Angina pectoris", "Sodium tanshinone IIA sulfonate", "Tanshinone IIA sulfonate", "randomized controlled trial", "randomized". The combination of subject word + free word is adopted, and adjusted according to different retrieval strategies of each database.

2.2 Inclusion criteria

2.2.1 The type of research

Western therapy or routine therapy? Author may need to standardize it's format.

STS combined with conventional Western therapy for RCTs of UAP;

2.2.2 Research object

According to the diagnostic criteria for unstable angina in the "Guidelines for Emergency Diagnosis and Treatment of Acute Coronary Syndrome (2019)", compliance with diagnostic criteria is sufficient, without limitation on age, gender, course of disease, and other conditions;

2.2.3 Intervention study

The control group was treated with Routine treatmen (RT), including regular rest, oxygen intake,nitric acid ester,β-receptor antagonists,antiplatelet agglutinating drugs, angiotensin converting enzyme inhibitors, calcium antagonists, and statins for treatment, and so on; The treatment group was treated with STS on the basis of the

control group;

2.2.4 Outcome indicators

The main outcome indicators are electrocardiographic efficacy (ECG), frequency of angina attacks, and duration of angina attacks,secondary outcome indicators are efficacy of clinical symptomsy (total effective = significant + effective), blood rheology (plasma viscosity,whole blood low viscosity,whole blood high shear viscosity;red cell aggregation index), blood lipid [total cholesterol (TC), triacylglycerol (TG), low-density lipoprotein cholesterol (LDL-c), high-density lipoprotein cholesterol (HDL-c)], C-reaction protein (CRP), adverse reactions.

2.3 Exclusion criteria

(1) Non-clinical randomized controlled trial;

(2) The interventions did not meet inclusion criteria, unclear diagnosis, or other serious diseases exist;

(3) The data is not rigorous or effective outcome indicator information cannot be extracted;

(4) Repeated publications, reviews, and animal experiments.

2.4 Data management and analysis

The two researchers(MXF and XQW) independently read and selected the full text of the literature and extracted relevant information, including the basic information of the literature (author, publication time), the number of patients, intervention methods (intervention measures, dose, duration), outcome indicators, adverse reactions, and other specific content. The two sides cross checked, and when

differences arise, they were referred to a third party (SNW) for discussion and evaluation, and information was extracted from the final literature.The included literature was evaluated based on the bias risk assessment tool for clinical randomized controlled trials in the Cochrane manual. The evaluation items involved random sequence generation methods, allocation concealment, blinding implementation of subjects and investigators, blinding implementation of outcome evaluators, incomplete outcome reports, selective reports, and other sources of bias. Each of the above items is judged by "low risk", "high risk", and "unclear".

2.5 Statistical analysis

Statistical analysis was conducted using RevMan5.3 software provided by the Cochrane system.The odds ratio (OR) is used as the combined statistic for counting data, and the mean difference (MD) is used as the combined statistic for measuring data. If different measurement methods or units are used, the standardized mean difference (SMD) is used as the combined statistic for measuring data, all expressed in 95% confidence interval (CI).The test for heterogenicity can be quantitatively judged by I2 and P values,When $P > 0.1$ and $I^2 \leq 50\%$, the fixed effect model is used for meta analysis, otherwise the random effect model is used for combined effect quantity analysis.At the same time, subgroup analysis was used to explore the source of heterogeneity in outcome indicators with large heterogeneity, and then sensitivity analysis or descriptive analysis was conducted through a one-by-one exclusion method. When the number of included studies was greater than 10, a funnel map was drawn to further assess whether there was publication bias.

Results

3.1 Results of the literature search

A total of 849 articles were retrieved, and 37 articles were finally included after screening[9-45],36 Chinese and 1 English, involving 3926 patients, including 2161 patients in the treatment group and 1765 patients in the control group. The literature screening process is shown in Figure 1, and the basic information for the included studies is shown in Table 1.

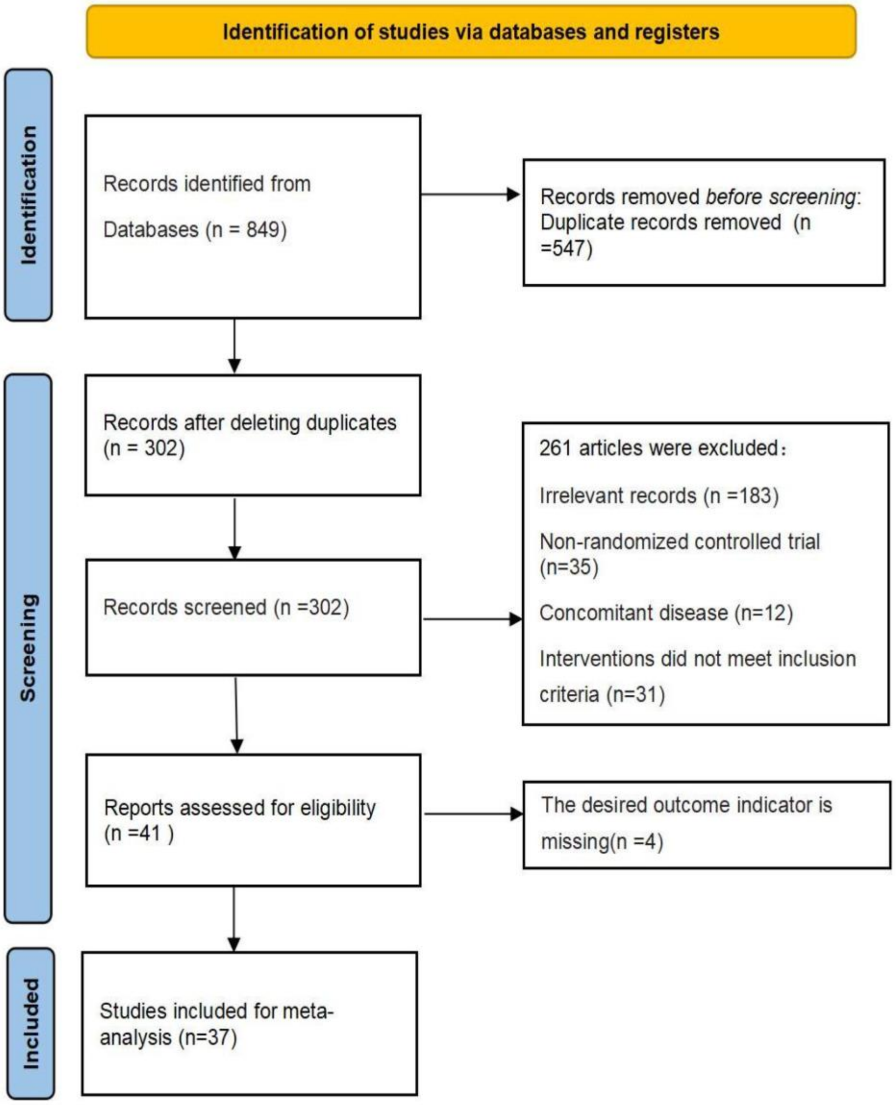

Figure 1 PRISMA flow-chart of the study selection process.

Table 1 Basic characteristic of include studies.

References	Group	Sample size	Interventions	Duration	outcome indicators	adverse reactions
Zhang Y 2013	T	83	RT+STS 60mg qd	14D	①②③④⑤⑥⑦⑧	Yes
	C	80	RT			
Li RJ 2017	T	40	RT+STS 80mg qd	14D	①②	No
	C	40	RT			
Li PQ 2012	T	40	RT+STS 70mg qd	14D	①④	No
	C	40	RT			
Xue Y 2012	T	40	RT+STS 50mg qd	14D	①	No
	C	40	RT			
Jin ZG 2016	T	50	RT+STS 60mg qd	14D	①②⑩⑪⑫⑬	Unclear
	C	50	RT			
Ju H 2013	T	30	RT+STS 60mg qd	10D	①④	Yes
	C	30	RT			
Liu ZH 2014	T	45	RT+STS 60mg qd	14D	①	Unclear
	C	45	RT			
Si QX 2012	T	566	RT+STS 80mg qd	14D	①②③	Yes
	C	201	RT			

Li DG 2017	T	48	RT+STS 40mg qd	10D	①⑤⑥⑦⑧	Unclear
	C	48	RT			
Huang LY 2013	T	40	RT+STS 30mg qd	14D	①	Unclear
	C	40	RT			
Zhao FL 2012	T	40	RT+STS 40mg qd	14D	①⑤⑥⑦⑨	No
	C	40	RT			
He F 2014	T	30	RT+STS 60mg qd	15D	①④⑨	Unclear
	C	30	RT			
Zhang ZH 2013	T	55	RT+STS 60mg qd	14D	①④	Yes
	C	55	RT			
Zou JM 2017	T	140	RT+STS 140mg qd	28D	①②	Unclear
	C	140	RT			
Du FD 2018	T	50	RT+STS 60mg qd	14D	①⑤⑧	Unclear
	C	50	RT			
Gao Y 2014	T	30	RT+STS 60mg qd	14D	①④⑨⑩⑪⑫⑬	Unclear
	C	30	RT			
Wang HY 2013	T	50	RT+STS 40mg qd	28D	①⑤⑥⑦⑨	Unclear
	C	50	RT			
Chen G	T	40	RT+STS 60mg qd	28D	①②③④	Yes

2013	C	40	RT			
Fan ZJ 2016	T	35	RT+STS 60mg qd	14D	①	Unclear
	C	35	RT			
Zhang HY 2014	T	40	RT+STS 60mg qd	14D	①⑨	No
	C	40	RT			
Li W 2012	T	42	RT+STS 40mg qd	14D	①	Yes
	C	36	RT			
Huang JN 2012	T	60	RT+STS 50mg qd	14D	①④⑪⑫⑬	Unclear
	C	60	RT			
Pei X 2009	T	36	RT+STS 40mg qd	14D	①⑤⑥⑦	Yes
	C	35	RT			
Wang JH 2010	T	38	RT+STS 60mg qd	14D	①④	Unclear
	C	38	RT			
Ge HZ 2010	T	30	RT+STS 60mg qd	15D	①④⑩⑪⑫⑬	No
	C	30	RT			
Yang N 2010	T	32	RT+STS 60mg qd	7D	①④⑤⑨	No
	C	32	RT			
Liu HL 2010	T	50	RT+STS 50mg qd	14D	①	No
	C	50	RT			
Huang H	T	53	RT+STS 60mg qd	14D	①④	No

2011	C	45	RT			
Luo WH	T	60	RT+STS 50mg qd	14D	①⑤⑥⑦⑧⑩⑪	No
2011	C	57	RT			
Song WJ	T	37	RT+STS 40mg qd	14D	①④	Unclear
2011	C	37	RT			
Zhang W	T	42	RT+STS 40mg qd	14D	①④	Unclear
2011	C	42	RT			
Chen XL	T	36	RT+STS 50mg qd	10D	①②③④	Yes
2008	C	28	RT			
Qin YW	T	35	RT+STS 40mg qd	15D	①④⑨	Yes
2008	C	34	RT			
Xu GP 2008	T	37	RT+STS 80mg qd	7D	⑨	Unclear
	C	37	RT			
Wang QL	T	25	RT+STS 50mg qd	14D	①④⑤⑧⑨	No
2007	C	25	RT			
Ma JH 2007	T	30	RT+STS 40mg qd	14D	①④	No
	C	29	RT			
Zhang HR	T	26	RT+STS 80mg qd	14D	①④	Yes
2006	C	26	RT			

Annotation 1:T:treatment group;C:control group; RT:Routine treatment including regular rest,

oxygen intake,nitric acid ester,β-receptor antagonists,antiplatelet agglutinating drugs, angiotensin converting enzyme inhibitors, calcium antagonists, and statins for treatment, and so on.

formatting or font issue?

Annotation 2:outcome indicators:①clinical efficacy;②frequency of angina attacks;③duration of angina attacks;④ECG;⑤plasma viscosity;⑥whole blood low viscosity;⑦whole blood high shear viscosity;⑧red cell aggregation index;⑨CRP;⑩TC;⑪TG;⑫LDL-c;⑬HDL-c.

3.2 Quality evaluation of literature

The Cochrane manual was used to assess the bias of the included literature. A total of 37[9-45]articles were included，2 studies[17、27]using a random number table method，1 study[28]using a computer-generated program randomization method，1 study[25]mentioning random grouping according to the order of patient visits，3 studies[13、36、39]mentioning random grouping according to the order of admission,and the remaining 30 articles mentioning random grouping but not specifically described，7 studies[13、17、25、27、28、36、39]have identified allocation considerations as high risk，2 studies[10、45]mentioned the use of single blinding,and the specific assessment information of bias risk included in various studies is shown in Tables 2,figure 2 and 3.

Table 2 Bias risk assessment findings incorporated into the study

References	Random sequence generation	Allocation concealment	Blinding of participants and personne	Blinding of outcome assessmen	Incomplete outcome data	Selective reporting	Anything else

				t			
Zhang Y 2013	U	U	H	H	L	L	U
Li RJ 2017	U	U	L	H	L	L	U
Li PQ 2012	U	U	H	H	L	L	U
Xue Y 2012	U	U	H	H	L	L	U
Jin ZG 2016	H	H	H	H	L	L	U
Ju H 2013	U	U	H	H	L	L	U
Liu ZH 2014	U	U	H	H	L	L	U
Si QX 2012	U	U	H	H	L	L	U
Li DG 2017	L	H	H	H	L	L	U
Huang LY 2013	U	U	H	H	L	L	U
Zhao FL 2012	U	U	H	H	L	L	U
He F 2014	U	U	H	H	L	L	U
Zhang ZH 2013	U	U	H	H	L	L	U
Zou JM 2017	U	U	H	H	L	L	U
Du FD 2018	U	U	H	H	L	L	U

Gao Y 2014	U	U	H	H	L	L	U
Wang HY 2013	H	H	H	H	L	L	U
Chen G 2013	U	U	H	H	L	L	U
Fan ZJ 2016	L	H	H	H	L	L	U
HY Zhang 2014	L	H	H	H	L	L	U
Li W 2012	U	U	H	H	L	L	U
Huang JN 2012	U	U	H	H	L	L	U
Pei X 2009	U	U	H	H	L	L	U
Wang JH 2010	U	U	H	H	L	L	U
Ge HZ 2010	U	U	H	H	L	L	U
Yang N 2010	U	U	H	H	L	L	U
Liu HL 2010	U	U	H	H	L	L	U
Huang H 2011	H	H	H	H	L	L	U
Luo WH	U	U	H	H	L	L	U

2011							
Song WJ 2011	U	U	H	H	L	L	U
Zhang W 2011	H	H	H	H	L	L	U
Chen XL 2008	U	U	H	H	L	L	U
Qin YW 2008	U	U	H	H	L	L	U
Xu GP 2008	U	U	H	H	L	L	U
Wang QL 2007	U	U	H	H	L	L	U
Ma JH 2007	U	U	H	H	L	L	U
Zhang HR 2006	U	U	L	H	L	L	U

Annotation :L:low risk;U:unclear;H:high risk.

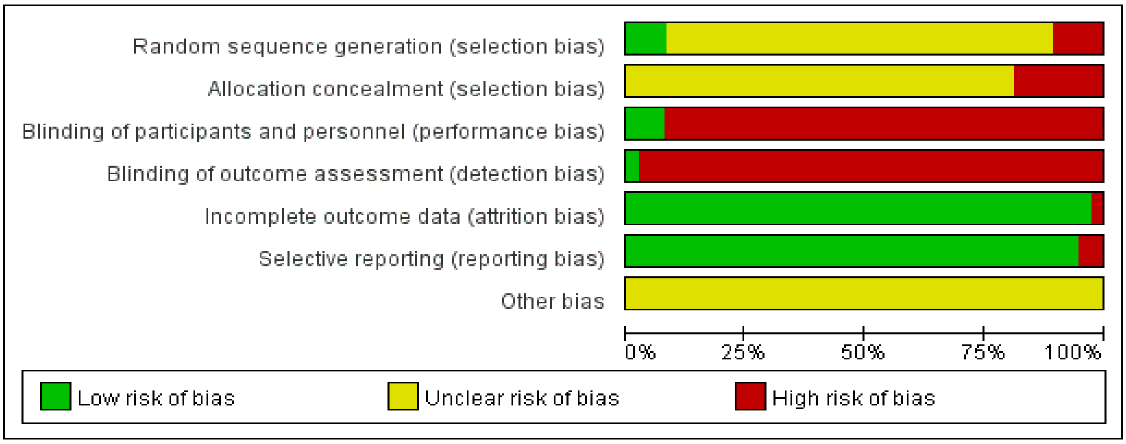

Figure 2 Bias risk assessment incorporated into the study

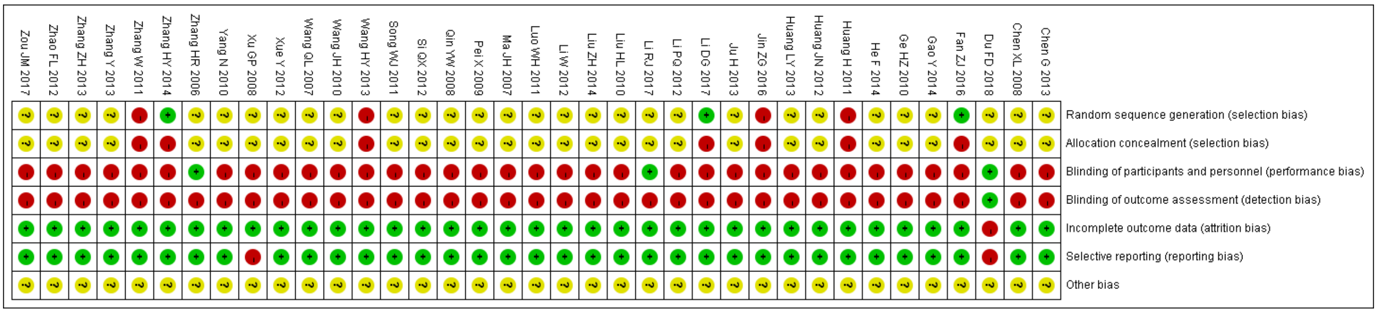

Figure 3 Bias risk assessment for individual studies

3.3 Meta analytic result

3.3.1 Main outcome indicators

(1)ECG efficacy:

19 studies[9、11、14、20、21、24、26、30、32-34、36、38-41、43-45]reported electrocardiographic efficacy, and heterogeneity testing showed that there was little heterogeneity between the studies (P = 0.91, I^2 = 0), using a fixed effect model for meta-analysis, the results showed that the difference was statistically significant (OR = 3.61, 95% CI [2.79, 4.68], P < 0.00001), indicating that the treatment group had better efficacy in improving patients' electrocardiograms than the control group, as shown in Figure 4.

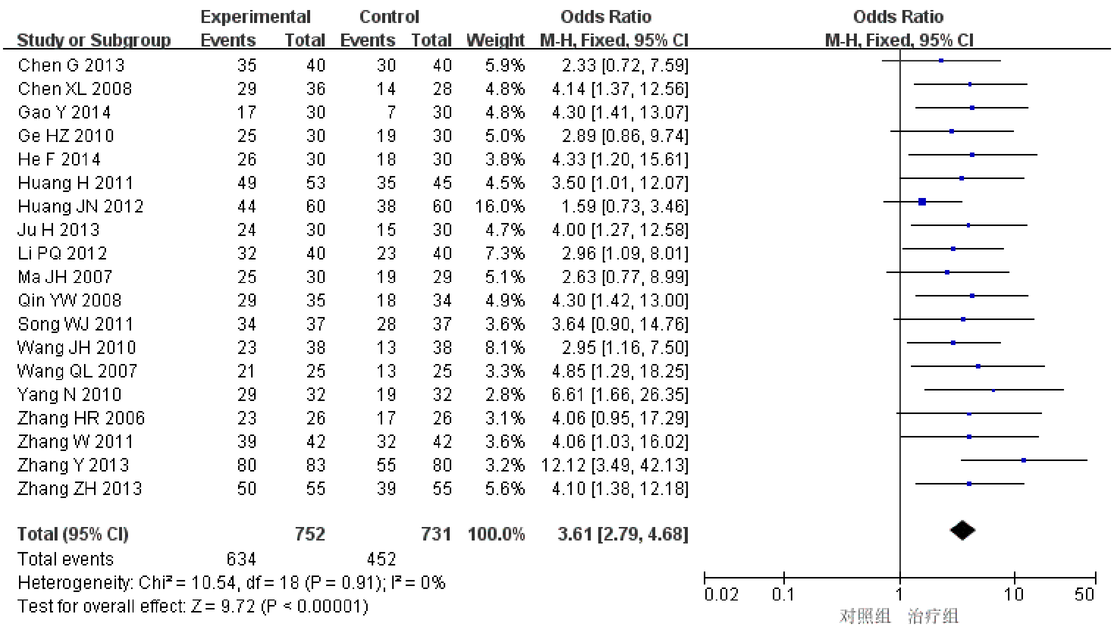

Figure 4 Meta-analysis of ECG efficacy

（2）Frequency of angina pectoris attacks:

7 studies[9、10、13、16、22、26、40]reported the frequency of angina pectoris attacks,and the heterogeneity test showed that there was significant heterogeneity between the studies (P < 0.00001, I^2 = 99%). Through subgroup analysis, sensitivity analysis, and reading the full text, no source of significant heterogeneity was found through factors such as dose and course of treatment. Therefore, a randomized response model was used for meta analysis, and the results showed that the difference was statistically significant (SMD=-2.61, 95% CI [-4.27, -0.96], P=0.002), this indicates that the treatment group has better efficacy in improving the frequency of angina attacks in patients than the control group, as shown in Figure 5.

Study or Subgroup	Experimental Mean	Experimental SD	Experimental Total	Control Mean	Control SD	Control Total	Weight	Std. Mean Difference IV, Random, 95% CI
Chen G 2013	3.5	0.4	40	5.9	0.5	40	13.9%	-5.25 [-6.19, -4.31]
Chen XL 2008	3.5	0.6	36	5.3	0.9	28	14.2%	-2.38 [-3.04, -1.73]
Jin ZG 2016	0.21	0.52	50	0.32	0.48	50	14.4%	-0.22 [-0.61, 0.18]
Li RJ 2017	2.18	1.02	40	4.29	1.07	40	14.3%	-2.00 [-2.54, -1.46]
Si QX 2012	2.7	0.4	566	5.1	0.4	201	14.4%	-5.99 [-6.34, -5.65]
Zhang Y 2013	2.1	1.1	83	3.8	1.6	80	14.4%	-1.24 [-1.57, -0.90]
Zou JM 2017	1.6	0.8	140	3.1	1.4	140	14.5%	-1.31 [-1.57, -1.05]
**Total (95% CI)**			**955**			**579**	**100.0%**	**-2.61 [-4.27, -0.96]**

Heterogeneity: Tau² = 4.92; Chi² = 690.80, df = 6 (P < 0.00001); I² = 99%
Test for overall effect: Z = 3.10 (P = 0.002)

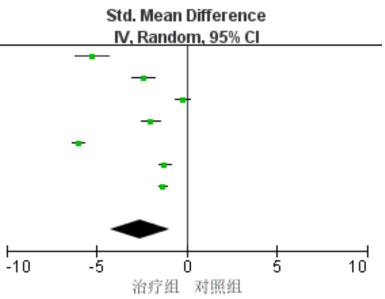

Figure 5 Meta-analysis off requency of angina attacks

（3）Time of angina attack:

4 studies[9、16、26、40] reported the onset time of angina pectoris, and the heterogeneity test showed significant differences among the studies ($P < 0.00001$, $I^2 = 98\%$). The results of meta-analysis using a random effect model showed that the differences were statistically significant (SMD = -4.01, 95% CI [-6.18, -1.84], P = 0.0003). Suggowed that the efficacy of the treatment group in improving the duration of angina attack was better than the control group, see Figure 6;Looking for the cause of high heterogeneity, through subgroup analysis of the treatment course, 2 articles[9、16]had a treatment course of 14 days, and found that the heterogeneity significantly decreased when the treatment course was 14 days ($P = 0.95$, $I^2 = 0$), with a statistically significant difference (SMD = -5.78, 95% Cl [-6.08, -5.48], $P < 0.00001$), indicating that STS was superior to the control group in reducing the onset time of angina pectoris;2 studies[26、40] with a non 14 days reported significant heterogeneity ($P = 0.002$, $I^2 = 89\%$), with statistically significant differences (SMD = -2.21, 95% Cl [-3.51, -0.92], P = 0.0008), indicating that STS has better efficacy in reducing the onset time of angina pectoris than the control group. The subgroup analysis results show that the treatment effect is better when the course of treatment is 14 days, as

shown in Figure 7.

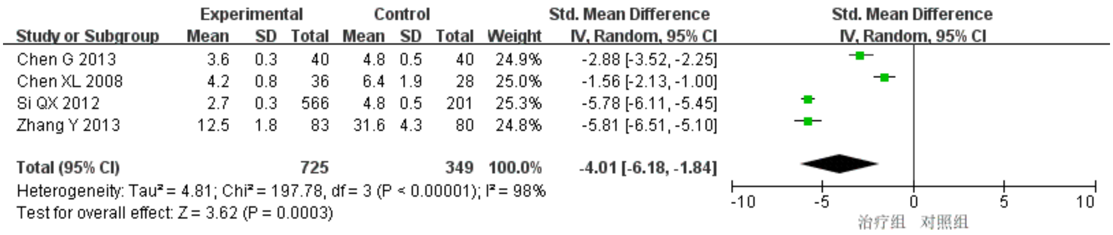

Figure 6 Meta-analysis of angina pectoris attack time

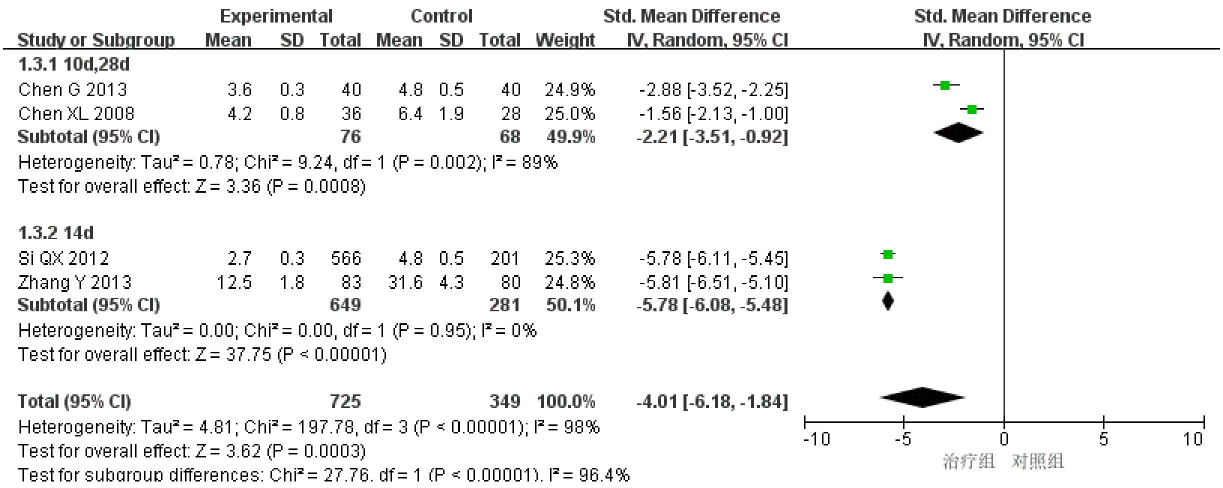

Figure 7 Subgroup analysis of angina pectoris attack time

3.3.2 Secondary outcome indicators

(1)Efficacy of clinical symptoms:

36 studies[9-41、43-45]reported clinical symptom efficacy, and the heterogeneity test showed that the heterogeneity between the studies was small (P = 1.00, I^2 = 0). Therefore, a fixed effect model was used for meta analysis. The results showed that the difference was statistically significant (OR = 4.02, 95% CI [3.32, 4.87], P < 0.00001), indicating that the treatment group had better efficacy in improving patients' clinical symptoms than the control group, as shown in Figure 8.

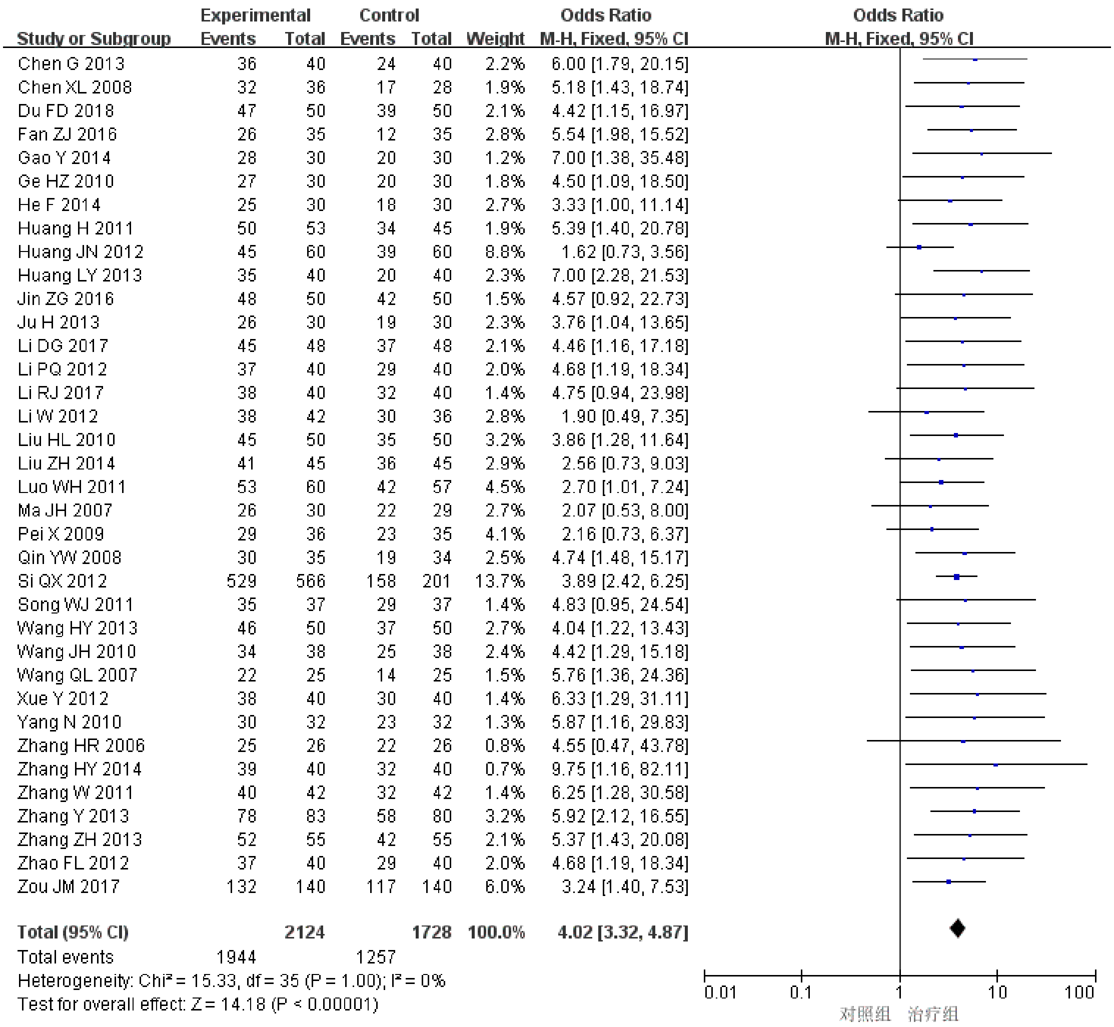

Figure 8 Meta analysis of efficacy of clinical symptomsy

（2）Blood rheology

9 studies[9、17、19、23、25、31、34、37、43]reported hemorheology, and the heterogeneity test showed that there was a large heterogeneity among the studies (P＜0.00001，I^2=90%)，the random effects model was used for meta-analysis, and the results showed that the difference was statistically significant (SMD=-0.93，95%CI [-1.20，-0.66]，P＜0.00001),the results showed that the treatment group was better than the control group in improving hemorheology；①Plasma viscosity：9 studies[9、17、19、23、25、31、34、37、43]reported plasma viscosity with high heterogeneity (P＜0.00001，I^2=93%)，so random effects model was used for meta-analysis. The results showed that: The

difference was statistically significant(SMD=-1.02，95%CI [-1.58，-0.47]，$P<0.0003$),indicating that the treatment group was better than the control group in improving plasma viscosity；②Whole blood low viscosity:6 studies[9、17、19、25、31、37]reported whole blood low viscosity with high heterogeneity (P=0.0003，I^2=79%)，the random effects model was used for meta-analysis, and the results showed that the difference was statistically significant(SMD=-0.85，95%CI [-1.21，-0.49]，$P<0.00001$),the results showed that the treatment group was better than the control group in improving the whole blood low viscosity ;③whole blood high shear viscosity:6 studies[9、17、19、25、31、37]reported whole blood high viscosity with high heterogeneity($P<0.00001$，I^2=91%)，the random effects model was used for meta-analysis, and the results showed that the difference was statistically significant(SMD=-0.82，95%CI [-1.44，-0.20]，P=0.009),the results showed that the treatment group was better than the control group in improving the whole blood high viscosity ;④Red blood cell aggregation：5 studies[9、17、23、37、43]reported red blood cell aggregation with high heterogeneity($P<0.00001$，I^2=94%)，the random effects model was used for meta-analysis, and the results showed that the difference was statistically significant(SMD=-1.00，95%CI [-1.75，-0.25]，P=0.009),the results showed that the treatment group was better than the control group in improving the red blood cell aggregation .Figure 9.

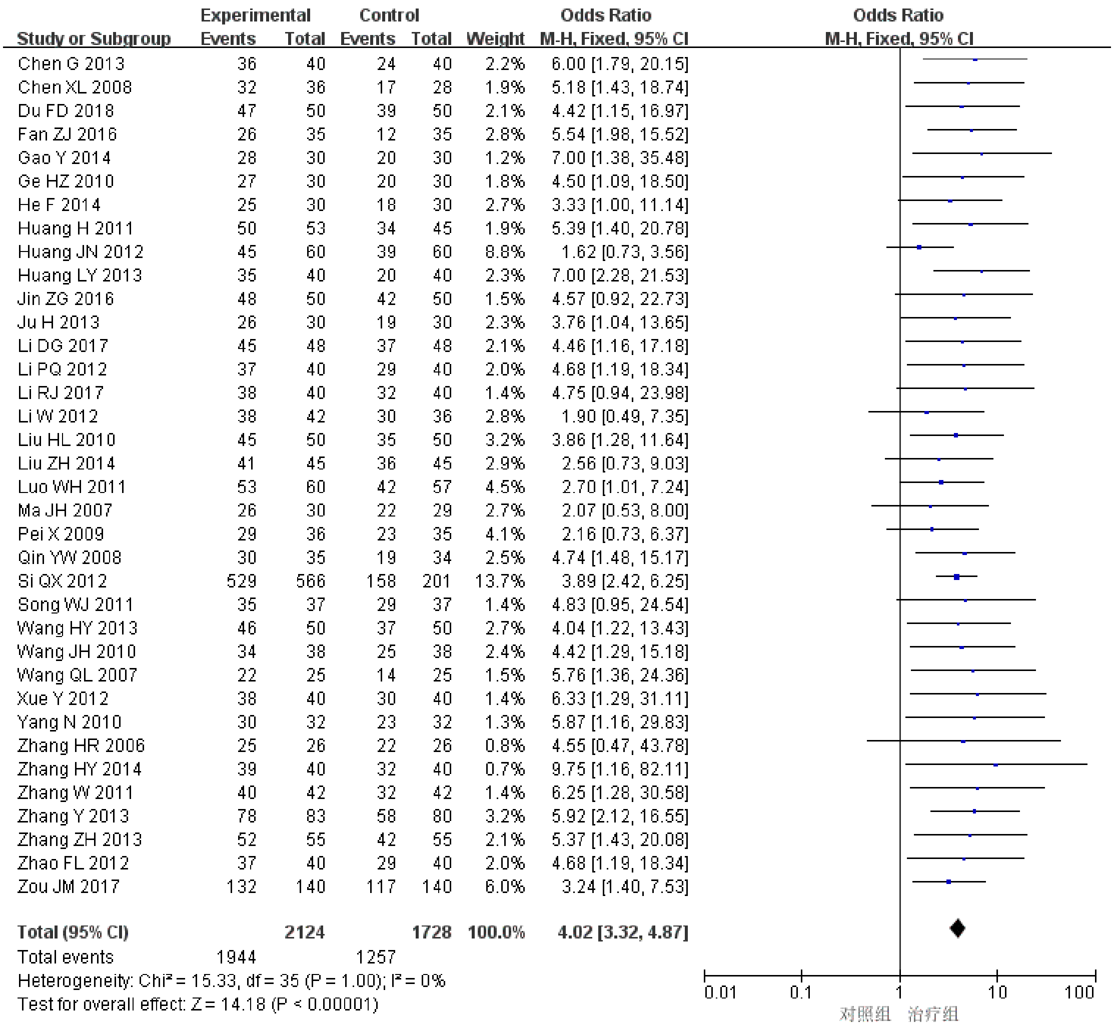

Figure 8 Meta analysis of efficacy of clinical symptomsy

（3）Blood lipid

5 studies[13、24、30、33、37]reported blood lipid with high heterogeneity(P＜0.00001，I^2=97%),the random effects model was used for meta-analysis, and the results showed that the difference was statistically significant(SMD=-1.15，95%CI [-1.88，-0.42]，P=0.002),the results showed that the treatment group was better than the control group in improving the blood lipid ;①TC：4 studies[13、24、33、37]reported TC with high heterogeneity(P ＜ 0.00001 ， I^2=98%),the random effects model was used for meta-analysis, and the results showed that the difference was statistically significant(SMD=-3.86，95%CI [-6.37，-1.34]，P=0.003)，the results showed that the

treatment group was better than the control group in improving the TC；②TG：5 studies[13、24、30、33、37]reported TG with high heterogeneity(P=0.0003，I^2=81%)，the random effects model was used for meta-analysis, and the results showed that the difference was statistically significant(SMD=-0.60， 95%CI [-1.04， -0.16]，P=0.008),the results showed that the treatment group was better than the control group in improving the TG;③LDL-c：4studies[13、24、30、33]reported LDL-c with high heterogeneity(P＜0.00001，I^2=95%)，the random effects model was used for meta-analysis, and the results showed that the difference was statistically significant(SMD=-1.54，95%CI [-2.67，-0.42]，P=0.007),the results showed that the treatment group was better than the control group in improving the LDL-c；④HDL-C：4studies[13、24、30、33]reported HDL-c with high heterogeneity,there was no significant difference between the treatment group and the control group(P=0.14＞0.05).Figure 10.

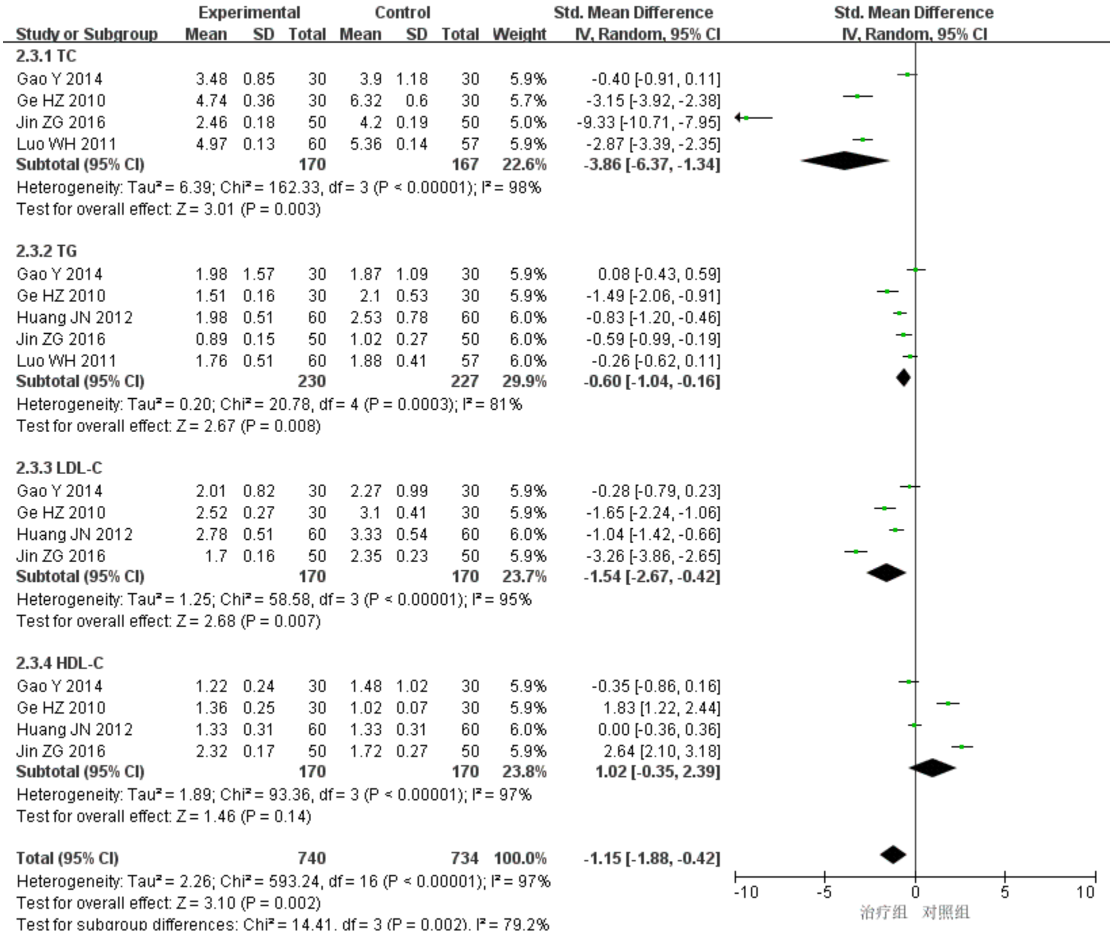

Figure 10 Meta-analysis of blood lipid

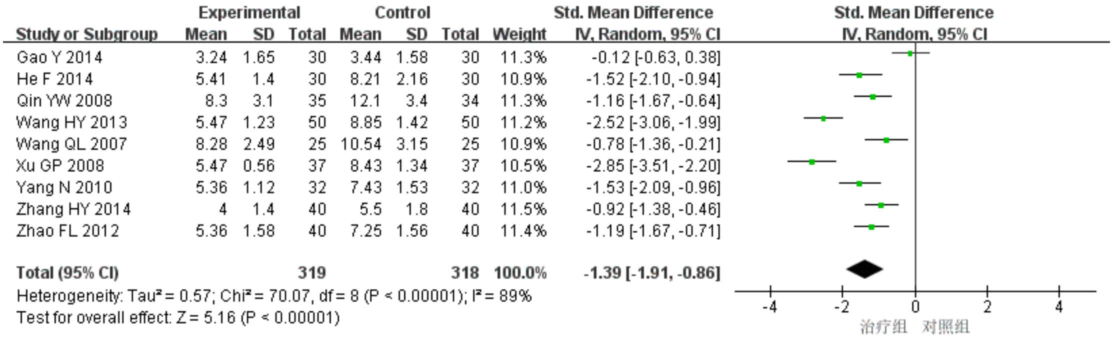

Figure 11 Meta-analysis of CRP

(4)CRP：9studies[19、20、24、25、28、34、41-43]reported CRP with high heterogeneity(P＜ 0.00001，I^2=89%)，the random effects model was used for meta-analysis, and the results showed that the difference was statistically significant(SMD=-1.39，95%CI

[-1.91，-0.86]，P<0.00001),the results showed that the treatment group was better than the control group in improving the CRP.Figure 11.

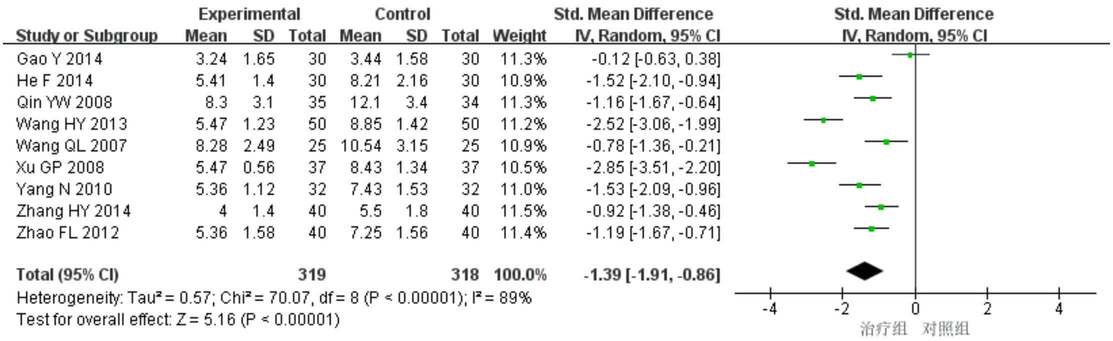

Figure 11 Meta-analysis of CRP

3.3.3 Adverse reactions

22 studies[9-12、14、16、19、21、26、28、29、31、33-37、40、41、43-45] report any adverse reaction in the

full stop, font error   confusing statement

process of the research。12 studies [10-12、19、28、33-37、43、44]report any adverse reaction in the process of the research,10 studies[9、14、16、21、26、29、31、40、41、45]reported adverse reactions, heterogeneity test, the heterogeneity between studies was small(P=0.56，I^2=0%)，so fixed effects model was used for meta-analysis, the results showed:the difference was statistically significant(OR=2.26，95%CI [1.06，4.85]，P=0.04)，indicating that the incidence of adverse reactions in the treatment group was higher than that in the control group. However, the adverse reactions in the two groups were not treated, and were alleviated by themselves after dropping rate was slowed down or drug withdrawal, which did not affect the study process. There were no significant changes in liver and kidney function before and after treatment in the two groups. The specific adverse reactions are shown in Table 3 and Figure 12.

Table 3 Adverse reactions

References	Treatment grou	Control group
Zhang Y 2013	2 cases had fever and 1 case had rash	None
Ju H 2013	1 case had mild headache.	None
Si QX 2012	5 cases of nausea, vomiting, occasional headaches	None
Zhang ZH 2013]	1 case of nausea, pain at the injection site.	None
Chen G 2013	None	1 case had headache and red face discomfort.
Li W 2012	None	3 cases of petechiae, ecchymoses subcutaneous injection site.
Pei X 2009	2 cases of red face with dizziness	None
Chen XL 2008	1 case of fever, 1 case of consciously headache, dizziness.	None
Qin YW 2008	3 cases of dizziness, 2 cases of facial flush	None
Zhang HR 2006	2 cases had swelling sensation in injection area	1 case of slight headache

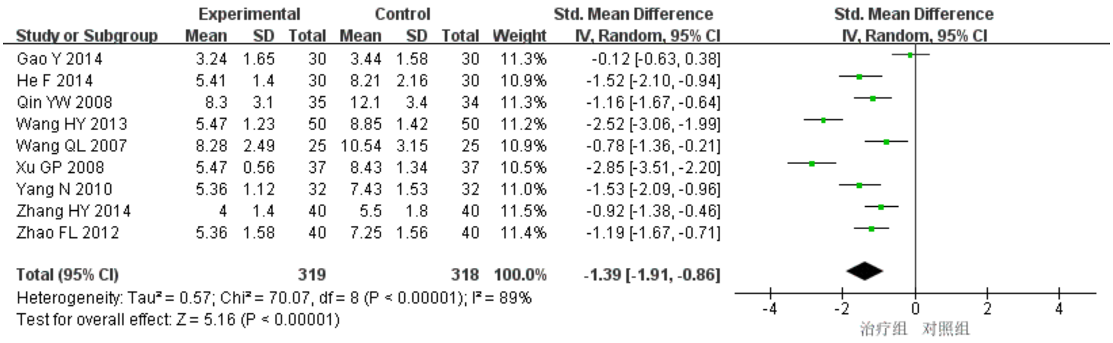

Figure 12 Meta-analysis of adverse reactions

3.4 Sensitivity analysis

Sensitivity analysis was conducted based on the meta-analysis results of TST in the treatment of UAP. All outcome indicators were removed one by one and no significant changes in outcome indicators were found in the re-analysis, indicating that the sensitivity of the overall analysis results was low and the outcome confidence was high.

3.5 GRADE quality evaluation

The results of this systematic evaluation were evaluated by the GRADE evidence quality grading system, which included bias risk, inconsistency, indirection, inaccuracy (-1 indicates serious, -2 indicates very serious) and publication bias (-1 indicates possible, -2 indicates very likely). Evidence quality was divided into four levels: high (-0), medium (-1), low (-2) and very low (-3). There are two levels of recommendation: strong and weak.The evidence quality of the relevant outcome indicators of TST treatment of UAP was mostly low and extremely low, and the recommendation strength was weak.Figure 13 and 14.

Outcome indicators for UAP

Patient or population: patients with UAP
Settings:
Intervention: Outcome indicators

Outcomes	Illustrative comparative risks* (95% CI) Assumed risk Control	Corresponding risk Outcome indicators	Relative effect (95% CI)	No of Participants (studies)	Quality of the evidence (GRADE)	Comments
**electrocardiographic efficacy**	**Study population**		**OR 3.61** (2.79 to 4.68)	1483 (19 studies)	⊕⊕⊖⊖ **low**[1,2,3,4,5]	
	**618 per 1000**	**854 per 1000** (819 to 883)				
	**Medium risk population**					
	**633 per 1000**	**862 per 1000** (828 to 890)				
**efficacy of clinical symptomsy**	**Study population**		**OR 4.02** (3.32 to 4.87)	3852 (36 studies)	⊕⊖⊖⊖ **very low**[3]	
	**727 per 1000**	**915 per 1000** (898 to 928)				
	**Medium risk population**					
	**731 per 1000**	**916 per 1000** (900 to 930)				
**frequency of angina attacks**		The mean frequency of angina attacks in the intervention groups was **2.61 standard deviations lower** (4.27 to 0.96 lower)		1534 (7 studies)	⊕⊖⊖⊖ **very low**[1]	SMD -2.61 (-4.27 to -0.96)
**duration of angina attacks**		The mean duration of angina attacks in the intervention groups was **4.01 standard deviations lower** (6.18 to 1.84 lower)		1074 (4 studies)	⊕⊖⊖⊖ **very low**[1]	SMD -4.01 (-6.18 to -1.84)
**Subgroup analysis of frequency of angina attacks - 10d,28d**		The mean Subgroup analysis of frequency of angina attacks - 10d,28d in the intervention groups was **2.21 standard deviations lower** (3.51 to 0.92 lower)		144 (2 studies)	⊕⊖⊖⊖ **very low**[1]	SMD -2.21 (-3.51 to -0.92)
**Subgroup analysis of duration of angina attacks - 14d**		The mean Subgroup analysis of duration of angina attacks - 14d in the intervention groups was **5.78 standard deviations lower** (6.08 to 5.48 lower)		930 (2 studies)	⊕⊕⊖⊖ **low**[1]	SMD -5.78 (-6.08 to -5.48)

*The basis for the **assumed risk** (e.g. the median control group risk across studies) is provided in footnotes. The **corresponding risk** (and its 95% confidence interval) is based on the assumed risk in the comparison group and the **relative effect** of the intervention (and its 95% CI).

CI: Confidence interval; **OR:** Odds ratio;

GRADE Working Group grades of evidence
High quality: Further research is very unlikely to change our confidence in the estimate of effect.
Moderate quality: Further research is likely to have an important impact on our confidence in the estimate of effect and may change the estimate.
Low quality: Further research is very likely to have an important impact on our confidence in the estimate of effect and is likely to change the estimate.
Very low quality: We are very uncertain about the estimate.

[1] Random method, allocation hiding, blind method and so on have production limitations
[2] Heterogeneity due to treatment course, dose and other factors
[3] The outcome index produces indirectness for the secondary index
[4] The confidence interval of the effect size is not narrow enough
[5] The funnel plot is asymmetric

Figure 13 GRADE quality evaluation of main outcome indicators

secondary outcome indicators for UAP

Patient or population: patients with UAP
Settings:
Intervention: secondary outcome indicators

Outcomes	Illustrative comparative risks* (95% CI) Assumed risk Control	Corresponding risk secondary outcome indicators	Relative effect (95% CI)	No of Participants (studies)	Quality of the evidence (GRADE)	Comments
**efficacy of clinical symptomsy**	**Study population**		**OR 4.02** (3.32 to 4.87)	3852 (36 studies)	⊕⊕⊝⊝ **low**	
	**727 per 1000**	**915 per 1000** (898 to 928)				
	**Medium risk population**					
	**731 per 1000**	**916 per 1000** (900 to 930)				
**blood rheology**		The mean blood rheology in the intervention groups was **0.93 standard deviations lower** (1.2 to 0.66 lower)		2521 (9 studies)	⊕⊝⊝⊝ **very low**[1]	SMD -0.93 (-1.2 to -0.66)
**blood rheology - plasma viscosity**		The mean blood rheology - plasma viscosity in the intervention groups was **1.02 standard deviations lower** (1.58 to 0.47 lower)		841 (9 studies)		SMD -1.02 (-1.58 to -0.47)
**blood rheology - whole blood low viscosity**		The mean blood rheology - whole blood low viscosity in the intervention groups was **0.85 standard deviations lower** (1.21 to 0.49 lower)		627 (6)		SMD -0.85 (-1.21 to -0.49)
**blood rheology - whole blood high viscosity**		The mean blood rheology - whole blood high viscosity in the intervention groups was **0.82 standard deviations lower** (1.44 to 0.2 lower)		527 (5)		SMD -0.82 (-1.44 to -0.2)
**blood rheology- red cell aggregation index**		The mean blood rheology- red cell aggregation index in the intervention groups was **1 standard deviations lower** (1.75 to 0.25 lower)		526 (5)		SMD -1 (-1.75 to -0.25)
**blood lipid**		The mean blood lipid in the intervention groups was **1.15 standard deviations lower** (1.88 to 0.42 lower)		1474 (5)		SMD -1.15 (-1.88 to -0.42)
**blood lipid - TC**		The mean blood lipid - TC in the intervention groups was **3.86 standard deviations lower** (6.37 to 1.34 lower)		337 (4)		SMD -3.86 (-6.37 to -1.34)
**blood lipid - TG**		The mean blood lipid - TG in the intervention groups was **0.6 standard deviations lower** (1.04 to 0.16 lower)		457 (5)		SMD -0.6 (-1.04 to -0.16)
**blood lipid - LDL-C**		The mean blood lipid - LDL-C in the intervention groups was **1.54 standard deviations lower** (2.67 to 0.42 lower)		340 (4)		SMD -1.54 (-2.67 to -0.42)
**blood lipid - HDL-C**		The mean blood lipid - HDL-C in the intervention groups was **1.02 standard deviations higher** (0.35 lower to 2.39 higher)		340 (4)		SMD 1.02 (-0.35 to 2.39)
**CRP**		The mean CRP in the intervention groups was **1.39 standard deviations lower** (1.91 to 0.86 lower)		637 (9)		SMD -1.39 (-1.91 to -0.86)
**New Outcome**	**Study population**		**RR 0** (0 to 0)	0 (0)	See comment	
	See comment	See comment				
	**Medium risk population**					

*The basis for the **assumed risk** (e.g. the median control group risk across studies) is provided in footnotes. The **corresponding risk** (and its 95% confidence interval) is based on the assumed risk in the comparison group and the **relative effect** of the intervention (and its 95% CI).

CI: Confidence interval; **RR:** Risk ratio; **OR:** Odds ratio;

GRADE Working Group grades of evidence
High quality: Further research is very unlikely to change our confidence in the estimate of effect.
Moderate quality: Further research is likely to have an important impact on our confidence in the estimate of effect and may change the estimate.
Low quality: Further research is very likely to have an important impact on our confidence in the estimate of effect and is likely to change the estimate.
Very low quality: We are very uncertain about the estimate.

[1] No explanation was provided

Figure 14 GRADE quality evaluation of secondary outcome indicators

3.6 Bias risk assessment

Risk of publication bias was assessed for outcome indicators of more than 10 articles included in this meta-analysis, and funnel plots of ECG efficacy and clinical

symptom efficacy were observed. It was found that the study sites were not completely symmetrical, suggesting the risk of publication bias. Figure 15 and 16.

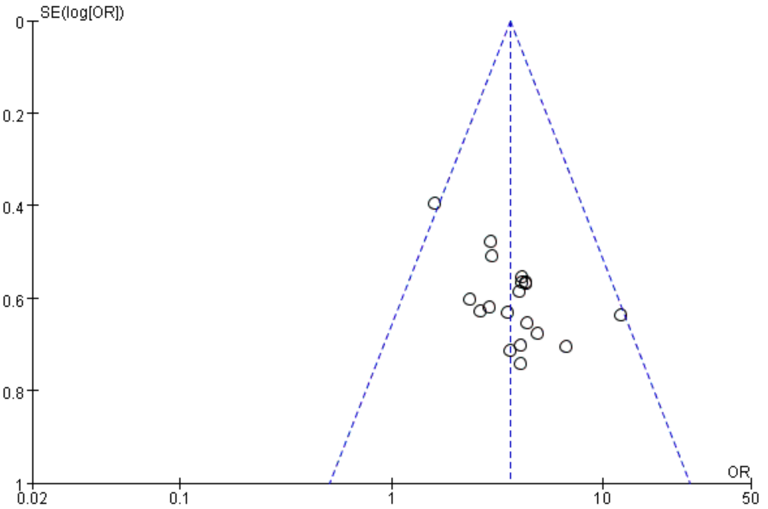

Figure 15 ECG efficacy funnel chart

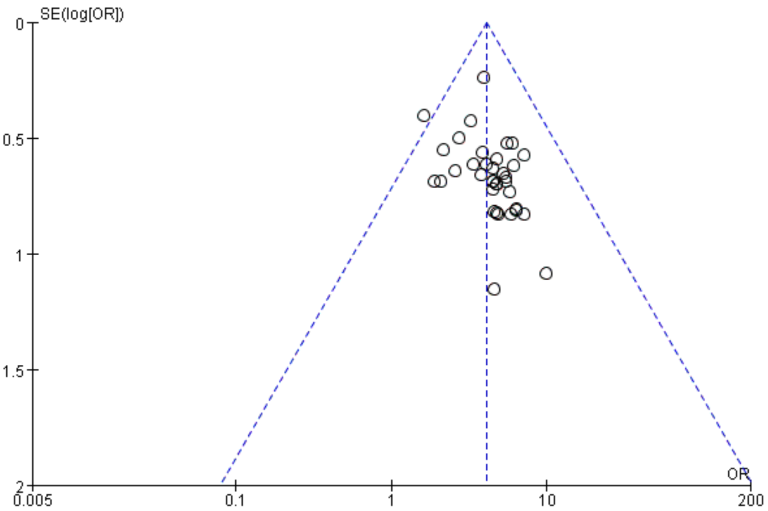

Figure 16 Clinical efficacy funnel chart

Discuss

4.1 Result analysis

Meta-analysis and systematic review have shown that the combination of TST

with routine treatmen can reduce the frequency and duration of angina attacks, improve ECG efficacy and clinical symptoms, reduce TG, TC, LDL-C, lower whole blood low shear viscosity, whole blood high shear viscosity, plasma viscosity and red blood cell aggregation. The effect of CRP reduction was superior to that of routine treatmen alone.As for the improvement of HDL-C, due to the few included studies and uneven quality, no ideal results have been obtained, which needs to be further confirmed in the literature. Subgroup analysis showed that 14 days of treatment was more effective in reducing the duration of angina attack. A few cases of adverse reactions occurred in the two groups, and the incidence of adverse reactions in the treatment group was higher than that in the control group. However, no abnormal liver and kidney function occurred in each study, which did not affect the research process.The clinical application of TST in the treatment of UAP is safe and effective, but the quality of the included literature is low. Therefore, higher quality randomized controlled clinical trials are needed in the future to further demonstrate the effectiveness and safety of TST and provide better clinical guidance.

4.2 Limitations

This study systematically evaluated the efficacy and safety of STS combination with routine treatme for UAP with the following limitations: ①Of the 37 studies included, only 2 were randomized with numerical tables, 1 was randomized with a computer-generated program, 1 was randomized in order of patient visit, 3 were randomized in order of admission, the remainder were randomized with only 7 high-risk randomized hidden studies, and only 2 were randomized with only partial

outcomes included in 37 studies that did not have full potential outcomes. ②The quality of the included literature is uneven, and the inclusion of age, sex, intervention course, and dose heterogeneity may also be important factors. ③None of the studies had long-term follow-up and there was a lack of long-term observation data.

4.3 Clinical significance

STS is mainly used in the clinical treatment of cardiovascular and cerebrovascular diseases, such as coronary heart disease and angina pectoris[46], which can effectively improve angina pectoris related indexes, optimize hemorheology indexes, improve blood lipid and reduce CRP leve[47].The significance of this systematic review mainly lies in the comprehensive collection of all Chinese and

extra spacing?

English literature on STS combined with routine treatmente in the treatment of UAP. After data screening and meta-analysis, a large-sample study result is finally obtained, which is more convincing and reliable than single-sample study [48]。

In order to further strengthen the evidence, future studies should develop rigorous implementation plans, design large sample, multi-center and long-term clinical randomized controlled trials based on evidence-based medicine standards, and pay attention to the correct and reasonable implementation of randomization, allocation scheme hiding and blind methods, so as to reduce selectivity, implementation, measurement and other biases. For the treatment of UAP by STS, different doses and courses of medication can produce different curative effects on patients. According to the use method of STS instructions and suitable population, unified standards of dosage and course of treatment should be formulated in the included studies. In

addition, the rate of drops should be controlled, with special attention paid to the allergic population, so as to improve the active treatment of adverse reactions in the treatment process. Record and report in detail to further improve the quality of evidence.[49]

Conclusion

In conclusion, based on the available evidence, the combination of STS with routine treatmen is effective and safe in the treatment of UAP. However, the overall quality of the literature included is low, and future clinical randomized controlled trials with higher quality and lower bias risk are needed to further analyze the efficacy and safety of STS and provide higher quality evidence for clinical work.

Ethical approval

Ethical approval and patient consent are not required since this is an overview based on published studies.

Funding Statement

Funding: This work was supported by the National Academic Schools of Traditional Chinese Medicine (XSLP-2013-35).

Conflicts of Interest

The authors declare that they have no competing interests.

Author Contributions

Xiaoqi Wu designed this study;Maoxia Fan and Xiaoqi Wu searched,screened, and extracted the information of the included studies, in dependently. Xiaoqi Wu and Maoxia Fan conducted the assessment.Sining Wei analyzed the data and wrote the

manuscript.Dong Guo revised the manuscript. All authors approved the final version for public acceptance, and all listed authors reviewed and revised the manuscript.

References

[1] Zhang X, Wu J, Zhang B, et al. Danshenchuanxiongqin injection in the treatment of unstable angina pectoris: a systematic review and Meta-analysis[J]. J Tradit Chin Med, 2016,36(2):144-150.

[2] Yeghiazarians Y, Braunstein J B, Askari A, et al. Unstable angina pectoris[J]. N Engl J Med, 2000,342(2):101-114.

[3] Mayer M. Anticoagulants in ischemia-guided management of non-ST-elevation acute coronary syndromes[J]. Am J Emerg Med, 2017,35(3):502-507.

[4] Wu J R, Liu S, Zhang X M, et al. Danshen injection as adjuvant treatment for unstable angina pectoris: A systematic review and meta-analysis[J]. Chin J Integr Med, 2017,23(4):306-311.

[5] Zhou Z Y, Zhao W R, Zhang J, et al. Sodium tanshinone IIA sulfonate: A review of pharmacological activity and pharmacokinetics[J]. Biomed Pharmacother, 2019,118:109362.

[6] Tan D, Wu J R, Zhang X M, et al. Sodium Tanshinone II A Sulfonate Injection as Adjuvant Treatment for Unstable Angina Pectoris: A Meta-Analysis of 17 Randomized Controlled Trials[J]. Chin J Integr Med, 2018,24(2):156-160.

[7] Zheng G. Expert suggestion on clinical application of tanshinone Ⅱ_A sodium sulfonate injection [J]. Chinese Journal of Cardio-Cerebrovascular Diseases in Geriatrics,2015,17(12):1261-1264.

[8] Page MJ, Moher D, Bossuyt PM, et al. PRISMA 2020 explanation and elaboration: updated guidance and exemplars for reporting systematic reviews. BMJ. 2021 Mar 29;372:n160.

[9] Zhang Yong, Cao Zhongchao, Liu Binsong. Clinical effect of tanshinone Ⅱ_A sodium sulfonate on unstable angina pectoris [J]. Journal of Inner Mongolia Medical University,2013,35(S2):382-384.

[10] Li Rongjun, Zhang Kuisong. Effects of tanshinone Ⅱ_A sodium sulfonate injection on platelet activation in patients with unstable angina pectoris syndrome [J]. Chinese Pharmaceutical Industry,2017,26(10):63-66.

[11] Li Peiqi, Wang Peng, Li Zhiqiang. Effect of tanshinone ⅡA sulfonate on myocardial ischemia in patients with unstable angina pectoris [J]. Chinese Medical Guide,2012,10(32):220-221.

[12] Xue Yan, Duan Hongli, Ren Juanhong. Clinical observation of 80 cases of unstable angina pectoris treated with tanshinone ⅡA sulfonate [J]. Jilin Medicine,2012,33(35):7694.

[13] Jin Zhankui. Therapeutic effect of tanshinone ⅡA sodium sulfonate on 100 cases of unstable angina pectoris [J]. Chin J Medica Guide,2016,14(23):177-178.

[14] JU H. Therapeutic effect of tanshinone ⅡA sulfonate on unstable angina pectoris [J]. Jilin Medical Journal,2013,34(08):1475.

[15] Liu Zhenhua. Clinical effect of tanshinone ⅡA sulfonate on unstable angina pectoris [J]. Clinical of Integrated Traditional and Western Medicine,2014,14(04):56-57.

[16] Si Qingxia. Effect of tanshinone ⅡA sulfonate on unstable angina pectoris of acute coronary syndrome in 566 patients [J]. Journal of Medical Forum,2012,33(12):56-57.

[17] Li Degang, Xu Daozhen. Clinical effect of tanshinone ⅡA sulfonate injection on unstable angina pectoris in senile patients and its improvement on hemorheology [J]. Modern Diagnosis and Therapy,2017,28(04):652-654.

[18] Huang Liya, Xu Kailian, Fu Yandao. Clinical effect of tanshinone ⅡA sulfonate injection on unstable angina pectoris [J]. Chin J Medical Guide,2013,11(33):498-499.

[19] Zhao Fenglin, Lu Xianbin, Yang Deqian, Huang Lumei. Clinical observation on the treatment of unstable angina pectoris by tanshinone ⅡA sodium sulfonate injection [J]. Chinese Emergency of

Traditional Chinese Medicine,2012,21(11):1844-1845.

[20] He Fei, Zhou Xinlang. Clinical effect of tanshinone ⅡA sulfonate injection on unstable angina pectoris [J]. Modern Practical Medicine,2014,26(07):815-816.

[21] Zhang Zhaohui, Zhang Hui. Clinical effect of tanshinone ⅡA sulfonate injection on unstable angina pectoris [J]. Chinese Journal of Disability Medicine,2013,21(03):108-109.

[22] Zou Jianming. Clinical effect of tanshinone ⅡA sulfonate injection on unstable angina pectoris [J]. Journal of Cardiovascular Diseases,2017,5(31):4-5+7.

[23] Du Fengdong. Effect of tanshinone ⅡA sulfonate injection on unstable angina pectoris of coronary heart disease [J]. Chinese medicine guidelines, 2018 (12) : 189-190. The DOI: 10.15912 / j.carol carroll nki gocm. 2018.12.151.

[24] Gao Yang, Liu Guangbin, Wang Jun. Treatment of unstable angina pectoris by tanshinone combined with nitrate [J]. Clinical Medicine Practice,2014,23(04):258-260.

[25] Wang Hongyan. Clinical observation of Tanshinone injection in treatment of unstable angina pectoris in elderly patients [J]. Western Chinese Medicine,2013,26(05):5-6.

[26] Chen G. Therapeutic effect of combined tanshinone ⅡA sulfonate injection on unstable angina pectoris [J]. Chinese Journal of Pharmaceutical Economics,2013(S3):63-64.

[27] Fan Zhengju, Yang Ming, Fu Yuan, Deng Hao. To explore the therapeutic effect of tanshinone ⅡA sulfonate injection on unstable angina pectoris of coronary heart disease and its effect on platelet activation function [J]. Chinese Journal of Biochemical Drugs,2016,36(03):140-142.

[28]Zhang H, Long M, Wu Z, Han X, Yu Y. Sodium tanshinone IIA silate as an add-on therapy in patients with unstable angina pectoris. J Thorac Dis. 2014 Dec; 6 (12) : 1794-9.

[29] Li W. Clinical observation of tanshinone ⅡA sulfonate combined with low molecular weight heparin

in treatment of unstable angina pectoris in senile patients [J]. Chinese Journal of Medical Sciences,2012,2(16):52-53+55.

[30] Huang Jingnan. Clinical observation on the treatment of unstable angina pectoris by tanshinone Ⅱa sulfonate injection [J]. Contemporary Medicine,2012,18(02):138.

[31] Pei Xing, Chen Gang. Treatment of unstable angina pectoris with tanshinone ⅡA sodium sulfonate combined with Western medicine in 36 cases [J]. Shaanxi Journal of Traditional Chinese Medicine,2009,30(10):1276-1278.

[32] Wang Junhui. Clinical study of tanshinone Ⅱ_A in the treatment of unstable angina pectoris [J]. Modern Distance Education of Chinese Traditional Medicine,2010,8(22):158-159.

[33] Ge Hai-zhu, CHENG Yao-nan, Qin Huan-yi. Clinical observation on the treatment of unstable angina pectoris with tanshinone ⅡA sulfonate [J]. Chinese Journal of Refractory Diseases,2010,9(03):201-202.

[34] Yang Ning, Ren Fuxian. Clinical application of tanshinone ⅡA sulfonate in the treatment of unstable angina pectoris [J]. Chinese Modern Doctors,2010,48(21):47-49.

[35] Liu Huiling, Yang Xiaohui. Effect of tanshinone ⅡA sulfonate on unstable angina pectoris [J]. Chinese Journal of Medical Innovation,2010,7(13):37.

[36] Huang He, Zhou Rong. Effect of tanshinone ⅡA sulfonate combined with isosorbide mononitrate on unstable angina pectoris [J]. International Medical Journal,2011,30(11):136.

[37] Luo Wanquan, Li Minli. Therapeutic effect of tanshinone ⅡA sulfonate on unstable angina pectoris [J]. Chinese Journal of Medical Sciences,2011,1(11):50+54.

[38] Song Weijun. Effect of tanshinone ⅡA sulfonate injection on unstable angina pectoris of coronary heart disease [J]. China Health Industry,2011,8(29):52.

[39] Zhang W. Therapeutic effect of tanshinone ⅡA sulfonate injection on unstable angina pectoris of coronary heart disease [J]. Chinese Journal of Modern Medicine,2011,18(35):72-73.

[40] Chen Xiaolong, Yang Jianzhong, Xiong Huisheng, Long Bo. Clinical study on the treatment of unstable angina pectoris of coronary heart disease with tanshinone ⅡA sulfonate [J]. Chongqing Medical Journal,2008(02):168-169+171.

[41] Qin Yuwang, He Xinbin, Zhang Zhenqian. Clinical effect of tanshinone ⅡA sulfonate injection on unstable angina pectoris [J]. Journal of Integrated Traditional Chinese and Western Medicine,2008(24):3768-3769.

[42] Xu Geping, Su Hua. Effect of tanshinone on inflammatory factors in patients with unstable angina pectoris [J]. Hainan Medicine,2008(03):31-33.

[43] Wang Ganlan, Deng Xiujuan, Li Xiaoqiu, Chen Xintong. Effects of tanshinone injection on CRP and D-dimer in patients with unstable angina pectoris [J]. New Chinese Medicine,2007(07):16-17.

[44] Ma Jianghua, Xiong Weiping, Wan Fengwei, Hua Mei. Treatment of unstable angina pectoris by tanshinone ⅡA sulfonate combined with Xinkang [J]. Chinese Modern Doctor,2007(14):97+105.

[45] Zhang Huarui, Sun Li, Li Yuchen, Jin Yukun. Effect of tanshinone Ⅱ_A sodium sulfonate injection on unstable angina pectoris [J]. Tianjin Pharmacy,2006(06):31-32.

[46] Zhang D, Wu J, Liu S, et al. Salvianolate injection in the treatment of unstable angina pectoris: A systematic review and meta-analysis[J]. Medicine (Baltimore), 2016,95(51):e5692.

[47] Xu J, Zhang C, Shi X, et al. Efficacy and Safety of Sodium Tanshinone IIA Sulfonate Injection on Hypertensive Nephropathy: A Systematic Review and Meta-Analysis[J]. Front Pharmacol, 2019,10:1542.

[48] Song H, Wang P, Liu J, et al. Panax notoginseng Preparations for Unstable Angina Pectoris: A

Systematic Review and Meta-Analysis[J]. Phytother Res, 2017,31(8):1162-1172.

[49] Fan Maoxia, Tian Ying, Guo Dong, Zhao Jisen, Liu Yongcheng. Efficacy and safety of Shugan Jieyu Capsule in treating essential hypertension and accompanying symptoms [J]. Hebei Journal of Traditional Chinese Medicine, 201,43(10):1728-1737.

Click here to access/download
Supporting Information
S1 Table.PRISMA 2020 Checklist 4.3.docx
